# Metabolite Diversity and Carbohydrate Distribution in *Brassica campestris* ssp. *chinensis* L. Cultivars: A UPLC-MS/MS Approach

**DOI:** 10.3390/biology13080568

**Published:** 2024-07-27

**Authors:** Hafiz Muhammad Mubeen, Ying Li, Chunmei Hu

**Affiliations:** National Key Laboratory of Crop Genetics & Germplasm Enhancement and Utilization, College of Horticulture, Nanjing Agricultural University, Nanjing 210095, China; muhammadmubeen732@gmail.com (H.M.M.); yingli@njau.edu.cn (Y.L.)

**Keywords:** Pak choi, non-heading Chinese cabbage (NHCC), metabolic analysis, metabolomics, carbohydrates, inter-cultivar comparison

## Abstract

**Simple Summary:**

Pak choi is a leafy green vegetable originating from China and is known for its nutritional value. It is important to understand which compound class is dominant and which cultivar contains the most nutritional value. The objective of the investigation was to ascertain how metabolic pathways influence the differences in the physiology, morphology, and nutritional value of different contents of Pak choi cultivars using advanced metabolomic technologies. A total of 513 metabolites fall into various classes, and subclasses were found, focusing on 16 key carbohydrates like simple sugars and complex carbohydrates. They found significant differences between the cultivars, with Suzhouqing having the most carbohydrates and Xiangqingcai having the least. These results are crucial for selecting the best cultivars for targeted metabolome, nutritional benefits, and cultivation. The study also revealed important metabolic pathways and highlighted that some cultivars are better suited for specific needs. By understanding these variations, this research supports better dietary choices and crop breeding strategies, contributing to improved nutrition and sustainable agriculture.

**Abstract:**

Pak choi exhibits a wide range of phenotypic and morphological variations, significantly impacting its carbohydrate composition. This study aimed to analyze these variations by employing UPLC-MS/MS technology on eight biological replicates of seven Pak choi cultivars. The untargeted metabolic analysis identified 513 metabolites, focusing on 16 key carbohydrates, including monosaccharides, disaccharides, and polysaccharides. Monosaccharides were the most prevalent, which were followed by di-, poly-, and oligosaccharides. Suzhouqing had the highest number of differentially accumulated metabolites (DAMs), while Xiangqingcai had the least. Notably, the cultivars Xiangqingcai, Suzhouqing, and Aijiaohuang showed significant metabolite differentiation. The study found 114 metabolites that differed significantly between Suzhouqing and Aijiaohuang, of which 69 were upregulated and 45 were downregulated. In Xiangqingcai and Aijiaohuang, 66 metabolites were upregulated and 49 were downregulated. Between Xiangqingcai and Suzhouqing, 80 metabolites were downregulated and 53 were upregulated. Key carbohydrate digestion and absorption pathways were identified alongside the most enriched flavonoid biosynthesis pathway in Xiangqingcai and Suzhouqing. The findings highlight the considerable carbohydrate variation among Pak choi cultivars, providing valuable insights for targeted carbohydrate extraction and improving nutritional and agricultural practices.

## 1. Introduction

Bok choy, also Pak choi or Chinese petiole cabbage, is a cultivar group of leafy vegetables that botanically belong to the Turnip species, but in everyday life they are more often called collard greens. The plants do not set heads, instead forming a dense rosette of smooth, dark green leaves with wide, fleshy petioles. Bok choy is popular in southern China and southeast Asian countries. Recently, it has been actively grown and sold in Europe and North America [1]. Previously, bok choy was distinguished as an independent botanical subspecies—*Brassica rapa* subsp. *chinensis* L., but this name is now considered an obsolete synonym for the species itself. With the modern approach, in accordance with the rules of the ICBN and ICND, the correct name for the group of varieties is *Brassica rapa* Pak Choi Group [2]. Pak choi and *Arabidopsis* share an intriguing relationship, as both belong to the Brassicaceae family. There are different varieties of bok choy including regular bok choy, milk bok choy, small bok choy, Shanghai bok choy, and Shanghai baby bok choy offering a platform for comparative analysis. Pak choi is notable for primary and secondary untargeted metabolites [3]. These compounds hold significance due to their potential roles in plant defense and environmental adaptation and their potential benefits for human health, as they play a central role in dietary management and prevent the risks associated with cardiovascular disease, diabetes, and obesity. One is vitamin C, which is a powerful antioxidant that protects cells from harmful radicals [4]. Vitamin C is vital in supporting immune function and may reduce the risk of infections. Pak choi is also an excellent source of vitamin K, essential for maintaining bone health, and may reduce the risk of osteoporosis [5]. It also contains glucosinolates, compounds with anti-inflammatory and anti-cancer properties, catering to health-conscious individuals. Meanwhile, *Arabidopsis*, a well-known plant model, possesses a vast gene pool of up to 25,500 genes and 107 distinct metabolite sequences [6]. The relevant metabolite sequences are confined to secondary metabolism. Secondary untargeted metabolites are abundant, and about 100,000 have already been identified, accounting for no more than 10% of all plant metabolites [7].

The carbohydrate metabolites derived from Pak choi, a nutritional gem within the *Brassica* family, hold immense significance for human health. Beyond being an energy source, these metabolites encompass many compounds that contribute to the vegetable’s health-promoting properties [8,9]. Carbohydrate metabolites in Pak choi, including dietary fiber and bioactive compounds, have been linked to various physiological benefits. They play a pivotal role in regulating blood sugar levels, offering potential advantages for individuals with diabetes and those striving for metabolic balance. Additionally, the diverse spectrum of metabolites, which extends to phytochemicals like glucosinolates and phenolic compounds, has shown promise in reducing the risk of chronic diseases. Their antioxidant, anti-inflammatory, and anti-cancer properties, among others, underscore their potential role in bolstering overall well-being [10]. Through thoroughly examining these carbohydrate metabolites and their mechanisms of action, we aim to shed light on the health benefits of Pak choi consumption, offering insights into how this vegetable can be a valuable addition to a health-conscious diet. Past research has involved the metabolic profiling of Pak choi leaves to elucidate the connection between the vegetable’s physical characteristics and metabolomic composition [11]. However, these prior studies may have specific limitations due to their focus on a limited number of cultivars. Hence, a compelling need arises for further investigations into the intricate relationship between Pak choi’s phenotype and metabolomic profile. Such comprehensive inquiries are essential for enhancing our understanding of how the plant’s appearance influences its metabolite composition and facilitating a more thorough evaluation of its nutritional value.

We randomly selected seven distinct Pak choi cultivars for this study and thoroughly examined their leaf metabolomic compositions. Initially, we applied an untargeted metabolomics methodology to appraise the complete spectrum of metabolites present in the leaf samples of Pak choi. Subsequently, we subjected the acquired dataset to multifaceted statistical analyses to explore potential correlations between the phenotypic traits of these cultivars and the content of metabolites. We then transitioned to a targeted analytical approach, aiming to fully comprehend the overall metabolic profile in the leaves of the diverse Pak choi cultivars.

## 2. Material and Methods

### 2.1. Plants and Chemical Agents

Seven cultivars of Pak choi, including Suzhouqing (SZQ), Yellowrose (YLR), Aijiaohuang (AJH), Wutacai (WTC), Xiangqingcai (XQC), Zicaitai (ZCT), and Ziluolan (ZLL), were employed in the experiment in 2023. First, plants are cultivated in plastic pots in a nutrient-rich, peat moss-and-soil-based medium with 2:1 and placed in a greenhouse. The internal factors of the greenhouse were fixed as daytime highs of 24 °C and 20 °C at night. The system’s relative humidity maintained between 75 and 80%. Pak choi was harvested at the age of six weeks when they attained some height in triplicates and were preserved right away in liquid nitrogen at −80 °C to denature hydrolytic enzymes, which turn the complex carbohydrates into simple compounds, as reported earlier [12]. The chemicals or reagents used in sample preparation were methanol, acetonitrile, and ethanol purchased from BioBioPa (http://www.biobiopha.com/, accessed on 12 May 2024). 

### 2.2. Standardization for Mass Spectrometry Analysis

For the precise quantification of metabolites in mass spectrometry, the standard solution purchased from Sigma-Aldrich (http://www.sigmaaldrich.com, accessed on 12 May 2024) was made with solvents like methanol or dimethyl sulfoxide (DMSO) that need cold storage at −20 °C to prevent deterioration and then diluted with an appropriate concentration of 70% methanol to a proper concentration (100 mg) before mass spectrometry analysis. 

### 2.3. Sample Preparation

The vacuum freeze drying of samples was carried out by maintaining the specific temperature and pressure so as not to affect the quality attributes. Experimental Mixer Mills MM 400 (https://www.retsch.com/, accessed on 12 May 2024) was used to powderize frozen specimens with a vibrational frequency of 30 Hz for 1.5 min. First, we weighed the powdered sample of 100 mg and dissolved it with 1.00 mL of extract in a vortex mixer at high speed, after which we vortexed it three times to maximize the extraction rate. Centrifugation followed at a speed of 10,000 RMP (revolutions per min) for 10 min to obtain the supernatant, and then filtration of the sample with a microporous filter of 0.22 μm porosity (SCAA-104, aperture size; ANPEL, Shanghai, China) (https://www.anpel.com.cn) accessed on 12 May 2024, after which it was kept in an injection tube for LC-MS/MS testing.

### 2.4. Factors of UPLC and Tandem Mass Spectrometry (MS/MS)

Data collection was performed by ultra-performance liquid chromatography (UPLC) (Shim-pack UFLC SHIMADZU CBM30A) (http://www.shimadzu.com.cn, accessed on 12 May 2024) and tandem mass spectrometry (https://sciex.com.cn) accessed on 17 May 2024. The standardized water conditions (weak solvent) in columns of UPLC (HSS T3 C18, (http://www.shimadzu.com.cn) accessed on 12 May 2024) were 1.8 μm and 2.1 mm × 100 mm of pure water with 0.04% acetic acid (Solute) with organic content of acetonitrile (strong solvent, + 0.04% acetic acid). There was a mobile phase with different concentrations, so gradient elution was performed for 0 min water/acetonitrile (95:5 *v*/*v*), 11:0 *v*/*v* for 5.95 min, 12:0 *v*/*v* for 5.95 min, 12:1 *v*/*v* for 95.5 min, 15:0 *v*/*v* for 95.5 min (*v*/*v* is the volumetric ratios). The stream rate was 0.4 mL/min with an injection volume of 2 μL, while the temperature of each column used in the system was 40 °C. Electrospray ionization (ESI) took place at a temperature of 500 °C, voltage of mass spectrometry of 5500 V, and the pressure of curtain gas (CUR) at 25 psi 3 [13]. The declustering potential (DP) and collision energy (CE) were used to analyze each ion pair in triple quadrupole (QQQ).

### 2.5. ESI-Q TRAP-MS/MS

An ESI-triple quadruple-linear ion trap (Q TRAP), API 6500 Q (www.appliedbiosystems.com.cn/ accessed on 12 May 2024), was acquired with LIT and a quadruple scans system. This system was accompanied by the ESI Turbo Ion-Spray interface, which was executed in positive charge configuration and administered by Analyst 1.6 software (AB SCIEX). The following specifications of ESI are the site of the ion, Turbo Spray, input temperature of 500 °C and 5500 V of ion spray voltage (IS), ion source gas I (GSI), gas II (GSII), curtain gas (CUR) at 55, 60, and 25.0 psi and potent collision gas (CAD).

In QQQ, similar LIT modes with 10 and 100 mol/L polypropylene glycol solution, equipment validation, and mass tweaking were executed. Investigations using multiple reaction monitoring modes (MRMs) with nitrogen (CAD) adjusted at a pressure of 5 PSI were used to obtain the scans of triple quadrupole (QQQ) and DP and CE optimization of MRM distinct shifts. A particular set of MRM flips during each interval was detected.

### 2.6. Analysis of Metabolites

Metabolomics investigates possible interactions and linkage between internal and external components in a cell, tissue, or organism. The metabolites were characterized according to their secondary spectral data, isotopic signal, and repeated signals containing K+, Na+, and NH4+, excluding persistent movements of ions with huge molecular weight. The metabolites were quantified employing the MRM configuration of triple quadruple mass spectrometry (QQQQ) (Supplementary Figure S1). Metabolite quantification was performed using QQQQ’s MRM. Its mechanism can be understood by the diagram (Supplementary Figure S2).

### 2.7. Analytical Stability of Data

For the stability and precision of analytical data, a quality control (QC) analysis is performed [14], and it is the method of standardization [15]. In QC, a sample is prepared to check the reproducibility of data by mixing all the samples and inserting them at the intervals of 10 tests. The repeatability and reliability of data are judged by mixing the ion flow patterns produced as a result of the spectrometry of QC samples. The signal stability of the instrument (MS) was ensured by analyzing the overlapping curves of metabolites.

### 2.8. Statistical Analysis

The complete set of statistical analyses, R 4.0.3 [16], was used for all data analysis. It considered principal component analysis (PCA), hierarchical cluster analysis (HCA), and fold change for eight biological replicates of each cultivar. All values were analyzed as mean ± standard deviation.

## 3. Results

### 3.1. Morphological and Phenotypic Variations in Pak Choi Cultivars

**Figure 1:**
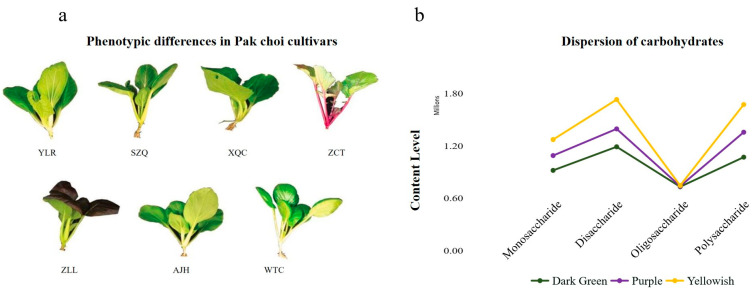
Phenotypic differences and carbohydrates classification in Pak choi cultivars (**a**) where YLR—Yellowrose, SZQ—Suzhouqing, XQC—Xiangqingcai, ZCT—Zicaitai, ZLL—Ziluolan, AJH—Aijiaohuang and WTC—Wutacai. (**b**) Division of carbohydrates based on leaf color.

For metabolic analysis, seven cultivars were grown under the conventional appropriate environmental conditions in the greenhouse. When harvesting, they showed differences morphologically and physiologically, which showed their specific cultivar character (Figure 1a). Although all the plants are grown under the same conditions, they show distinct variations in leaf color (green, dark green, pink, violet), leaf shape (oval and obovate), plant height (20–70 cm), leaf stalk color (green, dark green, violet) and growing pattern, as presented in Table 1.

By considering this, seven cultivars were classified into three groups based on their leaf color such as dark green (WTC, XQC), purple (ZLL), and yellowish (AJH, SZQ, ZTC, and YLR). The subclass of carbohydrates, i.e., monosaccharides, was determined to remain dominant in the dark green cultivar, which was followed by yellowish and then purple (Figure 1b and Table 2).

Five cultivars in the yellowish category have the highest disaccharides and fewer in purple and dark green, respectively. The polysaccharides and oligosaccharides were relatively similar across all cultivars, with their highest levels in dark green.

### 3.2. Metabolic Profiling and Principal Component Analysis of Metabolites (PCA)

**Figure 2:**
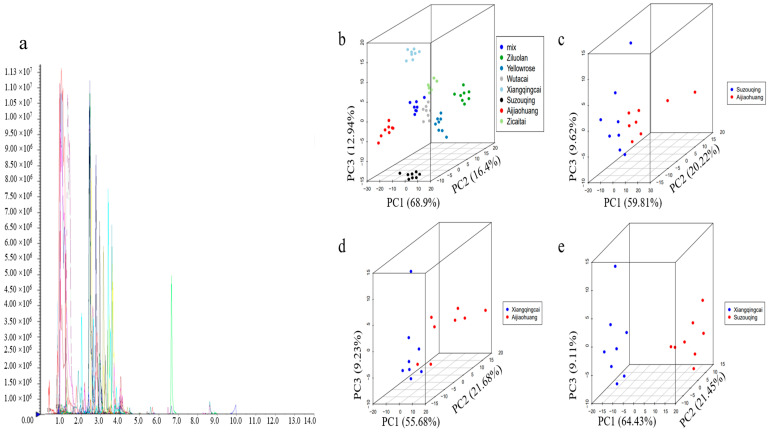
Detection and representation of metabolites. (**a**) The multi-peak plot of MRM detected metabolites, (**b**) principal component analysis (PCA) showed the metabolic profiling of all replicates, (**c**) PCA of SZQ and AJH, (**d**) PCA of XQC and AJH, and (**e**) PCA of XQC and SZQ.

The metabolites in each comparative group were quantified and identified using the MWDB database (https://www.metware.cn/ (accessed on 5 June 2024)). The triple quadrupole mass spectrometer was used to selectively screen for the target metabolites’ characteristic precursor and product ion pairs (transitions). The detector measured these characteristic ions’ signal intensity (counts per second, CPSs), as shown in Figure 2a. Principal component analysis (PCA) is very effective in converting the complex structures of all the metabolites to a more specific and targeted metabolite. Employing a model based on the PCA, an examination was conducted to scrutinize the quality and inherent variations among different metabolites. The PCA scores were observed to be dispersed across all samples, and further quality control (QC) measures were implemented to group all samples. This facilitated the detection of significant differences between each group and allowed for a more comprehensive and in-depth assessment of the natural variations among the diverse metabolites.

PCA of the seven cultivars with QC samples was conducted to obtain the profiling of the targeted metabolite (Figure 2b). The X-axis indicates the first principal component, the Y-axis shows the second principal component, and the third principal component is on the Z-axis. Group PCA was conducted to examine the differences between groups and specimens. The results revealed the dominant source of variation in PC1, which was 68.9% of the metabolic content of all the cultivars of Pak choi, especially in XQC, SZQ, and AJH, because of their Mahalanobis distances (2.78665, 1.0169 and 0.91243), which are far away from the mix (control), i.e., 0.01708. Because of the large number of cultivars and the quantitative differences based on their phenotype, we focused on these three cultivars and their interrelations with each other for further in-depth study. The PC1 of SZQ and AJH was 59.81%, while XQC and AJH were 55.68%, and XQC and SZQ were 64.43% (Figure 2c–e). In every analysis, the PC1 was more significant than 50%, showing that the division of metabolites or grouping is accountable, which means these cultivars were typical and specific.

### 3.3. Identification of Differentially Accumulated Metabolites

We employed the LC-MS approach, using eight biological replicates for each sample in a highly exhaustive manner, thereby leading to a comprehensive analysis of all metabolic profiles under investigation. In this analysis, we found 513 metabolites in total within 24 classes. Eleven groups belong to the primary metabolites, the most important of which are lipids, carbohydrates, and proteins. In contrast, 13 groups belong to secondary metabolites, such as vitamins, phytohormones, anthocyanins, alkaloids, terpenoids, flavonoids, etc., while 16 compounds are found in carbohydrates (Supplementary Table S1).

**Figure 3:**
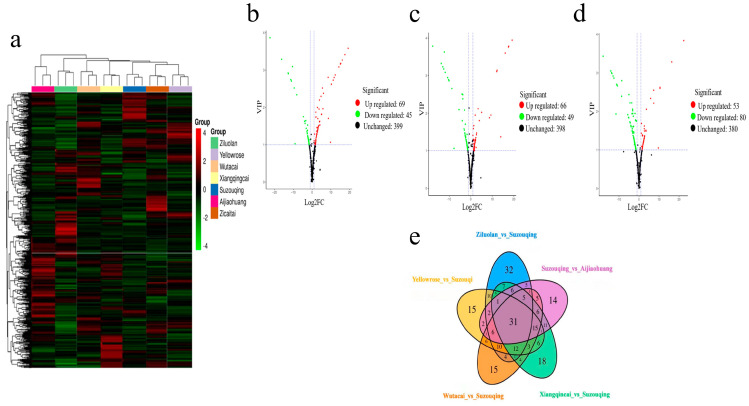
(**a**) Representation of metabolite detection. Volcano plots representing differentially expressed metabolites based on fold change value. (**b**): SZQ and AJH (**c**): XQC and AJH and (**d**): XQC and SZQ. (**e**) Venn diagram shows the relationship of DAMs in comparative groups.

Utilizing a hierarchal clustering approach, an evaluation was conducted to scrutinize the accumulation pattern of differential metabolites with their expression levels across all samples. Notably, the ZCT exhibited a more pronounced accumulation of metabolites with more elevated expression levels than the other cultivars examined, which was followed by AJH and SZQ, as represented in Figure 3a.

Orthogonal partial least squares-discriminant analysis (OPLS-DA) can measure less correlation between independent variables at X and dependent variables at Y. It increases the differentiation between groups and facilitates the quantification of specific metabolites. OPLS-DA combines orthogonal signal correction (OSC) and PLS-DA methods that convert the X-matrix data into two arrays uncorrelated to Y by removing the absurd differences. According to [17], the values of each subgroup were plotted to illustrate the differences between them.

Metabolites in the volcano plot represent each metabolite of the sample, the X-axis denotes the value of the logarithm of the quantification factor of a metabolite in the two samples, and the X-axis signifies the variable importance in the project (VIP) value. The greater the X value, the bigger the distinction between two expressions in two samples because of the log2 value of fold change. The more extensive the ordinates, the greater the significance of differential expression and the greater the accuracy of differentially expressed metabolites. It shows that compared to SZQ and AJH, there are 114 metabolites, out of which 69 were upregulated and 45 were downregulated (Figure 3b) (Supplementary Table S2).

The downregulated metabolites are shown in green, while upregulated metabolites are shown in red, and undifferentiated metabolites are shown in black. The more Y-vale represents, the more significant the differences between metabolites. XQC and AJH contributed a total of 115 metabolites with 66 upregulated and 49 downregulated (Figure 3c). In comparison, the third pairwise analysis of XQC and SZQ contributed a total of 133 metabolites, out of which 53 were upregulated and 80 were downregulated (Figure 3d).

The demonstration of the relationship between groups of differential metabolites is omitted here. A total of 115 metabolites are typical and found in all the cultivars used for the study (Figure 3e). The profiling of these groups shows that YLR and SZQ have 15, ZLL and SZQ have 32, SZQ and AJH have 15, ZLL and SZQ have 32, SZQ and AJH have 14, and WTC and SZQ have 15.

**Figure 4:**
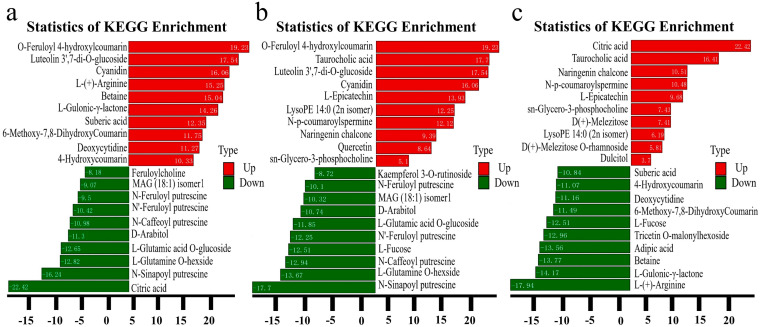
Analysis of the fold difference of metabolites between different comparative groups (**a**) SZQ and AJH, (**b**) XQC and AJH, (**c**) XQC and SZQ.

Following the descriptive analysis of the identified metabolites, a comparison was made regarding alteration in the multiplier of the metabolite quantification information across each group, considering the grouping of the specific sample. As shown in Figure 4, the FC of SZQ and AJH was 19.23 (Figure 4a), while XQC and AJH exhibited an FC value of 16.73 (Figure 4b). At the same time, the highest FC was found in XQC and SZQ with a value of 22.42 (Figure 4c).

It can be inferred that the differentiation of metabolites within or between groups is contingent upon the cultivar type. Thus, the cultivar’s phenology significantly impacts the number of metabolites.

### 3.4. Clustering of Differentially Expressed Metabolites

**Figure 5:**
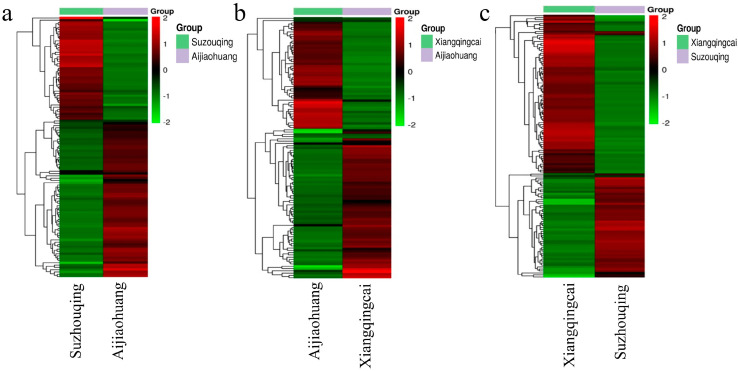
Clustering of differentially expressed metabolites in a combination of different cultivars of NHCC, including (**a**) SZQ and AJH, (**b**) XQC and AJH, and (**c**) XQC and SZQ.

Identifying clusters of metabolites that exhibit similar expressions across pairwise groups was studied. It was found that several metabolites showed the upregulation and downregulation differently in each group of cultivars. Upon analyzing SZQ and AJH, it was shown that out of the total metabolites, 114 exhibited substantial expression, with 58 showing an upregulated response (UPR) and 68 displaying a downregulated response (DNR). The overall metabolites, including flavonol, organic acids, and carbohydrates, were expressed in varying quantities (Figure 5a). SZQ made the most significant contribution to organic acids, specifically adipic acid, accounting for 60% of this category. On the other hand, AJH demonstrated a substantial contribution of 66% in carbohydrates.

The analysis of XQC and AJH exhibits distinct differences in their compounds, indicating a metabolic difference. A total of 115 metabolites were identified, with the majority (66) belonging to the UPR metabolites group, while 49 were classified as DNR (Figure 5b). The primary categories making the most significant contribution were amino acids and derivatives (AADs) and lipids, accounting for 56% of AADs from XQC with citric acid and 44% from AJH with compound glutaric acid B. In both XQC and AJH cultivars of Pak choi, lipids were the predominant compound, accounting for 51% and 49%, respectively. When examining the pairwise metabolic interaction between XQC and SZQ, it was observed that 133 out of 513 metabolites showed similarity in these cultivars, making them the most significant contributors to the interaction (Figure 5c). The primary metabolites contributing were organic acids, flavanol, carbohydrates, and AAD. These subclasses had 15, 14, 13, and 12 metabolites, respectively, whereas the remaining 79 came from 22 other secondary metabolites.

### 3.5. Kyoto Encyclopedia of Genes and Genomes (KEGG) Enrichment and Functional Annotation of Differential Metabolites

**Figure 6:**
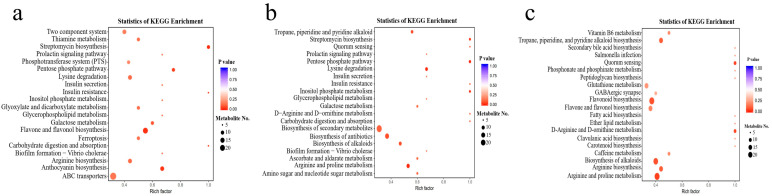
KEGG annotations and enrichment of differentially expressed upregulated metabolites where (**a**) SZQ and AJH, (**b**) XQC and SZQ, and (**c**) XQC and SZQ. Abscissa represents the quantification of metabolites, while ordinate shows the KEGG metabolic pathway found in the specific division as per order. **Note:** The rich factor is the ratio of the differentially expressed metabolites in the corresponding pathway to the total number of metabolites detected by the pathway. The higher the value, the greater the degree of enrichment. The closer the *p*-value is to 0, the more significant the enrichment. The size of the mid-point represents the number of metabolites enriched into the corresponding pathway with significant differences.

According to the KEGG analysis, carbohydrates have some critical pathways: glycolysis, the pentose phosphate pathway, galactose metabolism, and the citrate cycle (TCA cycle) are significant, utilizing the KEGG database to illustrate many metabolites in these assays. In KEGG enrichment of specific classification, the analysis of SZQ and AJH and XQC and SZQ (Figure 6a,b) showed the pathway of carbohydrates digestion and absorption with a total compounds of 243 out of which two were the most unique compounds, D-Glucose 6-phosphate and D(+)-Glucose, found in this pathway (Supplementary Figure S3). On the other hand, the most enriched pathway in XQC and SZQ (Figure 6c) was flavonoid biosynthesis with the contribution of seven unique compounds, including L-epicatechin, delphinidin, 7,4′-dihydroxyflavone, quercetin, naringenin chalcone, prunin, and cyanidin (Supplementary Figure S4).

### 3.6. Descriptive Analysis of Carbohydrates

**Figure 7:**
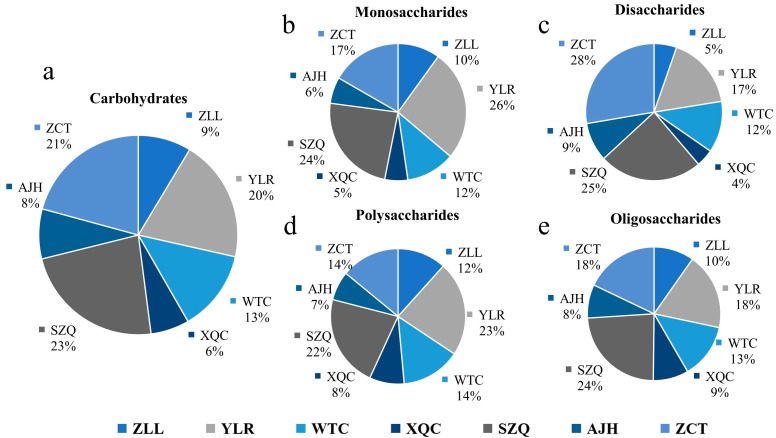
Differentiation of carbohydrates and subclasses in each cultivar of Pak choi based on the number of compounds against (**a**) total carbohydrates, (**b**) monosaccharides, (**c**) disaccharides, (**d**) polysaccharides (**e**) oligosaccharides.

An aggregate of 16 carbohydrate metabolites has been identified in the seven cultivars of Pak choi, with eight biological replicates of each, unevenly spread among numerous cultivars (Supplementary Table S3). Carbohydrates were categorized quantitatively based on their nature, such as monosaccharide, disaccharide, polysaccharide, and oligosaccharide (Figure 7a). The highest amount of carbohydrates was found in the ZCT cultivar, contributing 21%, while the lowest was by XQC. It showed that ten detected compounds of carbohydrates belong to a monosaccharide group, which is the highest number of compounds contributing in this category with a total of 26% by YLR. At the same time, the lowest was 6% by AJH (Figure 7b).

**Figure 8:**
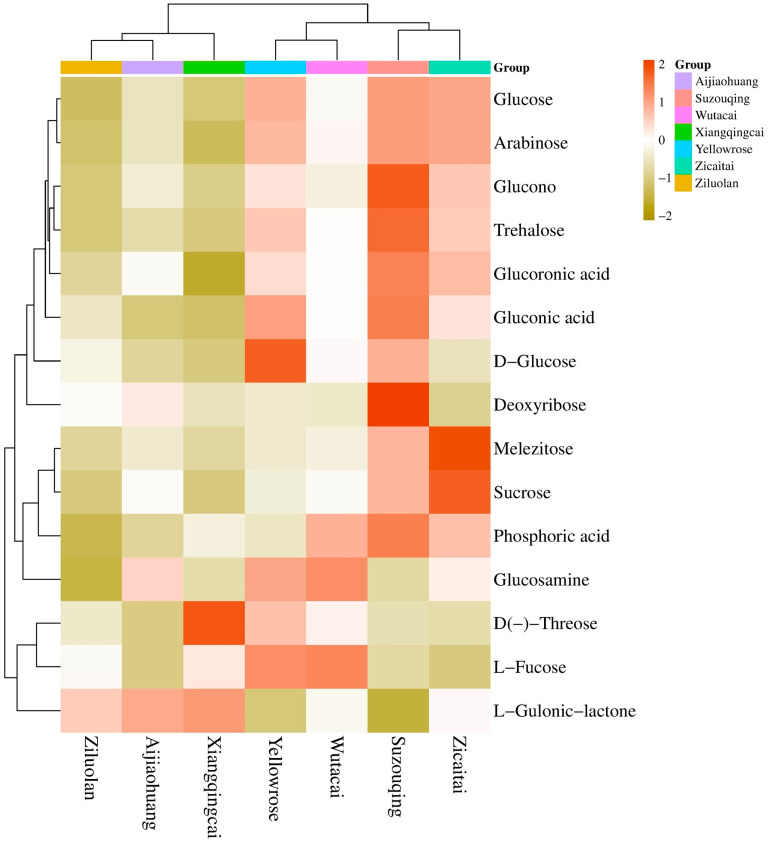
The expression level of carbohydrate compounds in different cultivars of Pak choi.

The three metabolites of carbohydrates belonging to this category, i.e., disaccharides, were found abundant in ZCT, while the lowest were in XQC, contributing 48% of the total carbohydrates in all the cultivars. In this classification, the most contributing compound is D-(+)-Sucrose, while the least was D (+)-Melezitose-O-rhamnoside (Figure 7c). Only two compounds of polysaccharides, including D-(+)-Glucono-1,5-lactone and Glucarate-O-Phosphoric acid, constitute 30% of the total carbohydrates. Most of the polysaccharides were found in cultivars in YLR, which was followed by SZQ and ZCT, respectively (Figure 7d). Oligosaccharides were minimal, with a maximum contribution from the cultivar SZQ and the lowest from AJH (Figure 7e). The common compound of carbohydrates found in the KEGG enrichment (Figure 6) was D-(+)-Glucose (C00031). Different expression levels of DAMs are shown in Figure 8. The heatmap shows that the Aijiaohuang cultivar has a high abundance of glucose, while the Xiangqingcai cultivar has a low abundance of glucose.

## 4. Discussion

Pak choi (*Brassica campestris* ssp. *chinensis*) is a very adaptable crop that matures quickly, produces high-quality leaves, has a distinct taste, and provides a wealth of nutrients; it originated in China [18]. This leafy green vegetable, bok choy, boasts a rich profile of vitamins, minerals, and bioactive compounds, making it a significant dietary component in various culinary traditions worldwide [19]. Carbohydrates are a fundamental class of macronutrients that serve as a primary energy source for the human body. These can be sourced from different ways, such as natural sources and synthetic sources. Plant-based carbohydrates are abundant in nature and serve as a primary energy source for humans. Carbohydrates are classified based on the amount and type of sugar molecules they contain, such as monosaccharides, disaccharides, polysaccharides, and oligosaccharides.

In the current study, 16 carbohydrate compounds were detected, of which the majority belong to the class of monosaccharides. The simplest form of carbohydrate consists of only one sugar unit or saccharide, a monosaccharide [20]. We have found that ten carbohydrate metabolites belong to the monosaccharide group, comprising 21% of the total carbohydrates. They play a central role in the biological processes of plants and animals, such as growth, nutrition, respiration transportation, etc. [21]. The most significant compound of monosaccharide was D-glucose 6-phosphate in YLR, which has a tremendous beneficial effect on human health in preventing brain tumors and helping in diabetic control [22]. The other important class of carbohydrates detected was disaccharides, featuring a combination of two monosaccharides that work as a source of energy in the human body and as a sweetener [23]. They play a central role in plants as transporters of nutrients to phloem and other cells for the different processes involved in the life cycle of plants [24]. These include maltose, lactose sucrose, etc. Disaccharides, the second most abundant class of carbohydrates, contain beneficial compounds such as D-(+)-Sucrose, which plays a central role in bloodstream regulation and acts as an energy storage and signaling molecule in plants [25].

According to previously reported studies, trehalose 6-phosphate is a crucial oligosaccharide that plays a central role in the developmental processes of plants and signaling molecules [26,27]. It was detected as minimal, with a combined contribution of only 1%, while it was maximum in SZQ and lowest in AJH. Polysaccharides are long-chain macromolecules made by many monosaccharides linked to each other through glycosidic bonds. Two compounds of this division found in our study were D-(+)-Glucono-1,5-lactone and Glucarate O-Phosphoric acid, which constitutes 30% of the total amount of carbohydrates where the maximum amount is found in YLR, which is followed by SZQ and ZCT, respectively. They are of great importance, as most of the carbohydrates are available in this form of polysaccharides. Their importance has already been proven by scientists, including [28,29], who found that polysaccharides sourced from Chinese medicines play a central role in the improvement of antibodies and the immune system as a vaccine against cancer and other lethal diseases.

In the analysis of most differentiated cultivars based on Mahalanobis distance, the results of PCA were XQC, SZQ, and AJH. The interrelation of these three cultivars was significant to study as it gives real insight into the metabolite differences. The study of metabolites and their interaction within the cultivars were studied in sweet potato [30], buckwheat [31], and olive [32].

ZCT exhibited a more pronounced accumulation of metabolites with elevated expression levels than the other cultivars examined, which was followed by AJH and SZQ. In the comparison of SZQ vs. AJH, the number of upregulated metabolites was 69, while 45 were downregulated. The most significant metabolites with increased abundance were O-feruloyl 4-hydroxycoumarin, luteolin 3′,7-di-O-glucoside, and cyanidin, with an FC range from 19.23 to 16.06. The metabolite that stands out the most is L-(+)-arginine with XQC with AJH, which showed a significant increase in abundance in XQC. The fold change for L-(+)-arginine is approximately 16.78. Arginine plays a role in several biological processes, such as protein synthesis, the urea cycle, and the creation of nitric oxide [33]. The notable rise indicates an improved level of metabolic activity in XQC. Significant enhancements in the former cultivar included adipic acid, cyanidin, L-gulono-y-lactone, quercetin O-acetylhexoside, and (+)-gallocatechin. The metabolites mentioned play crucial roles in many pathways, such as adipic acid in fatty acid metabolism [34], cyanidin and quercetin derivatives in antioxidant activity [35], and L-gulono-y-lactone in carbohydrate metabolism [36].

The metabolite that shows the most notable decrease in abundance in AJH is L-cysteine with a fold change of approximately −7.57. This decline is further surpassed by N-feruloyl putrescine, which exhibits a fold change of −9.7. Putrescine is a polyamine that has a role in cellular development and stress response. The decrease in AJH may suggest that there have been changes in the development and metabolic functioning of Pak choi versus AJH. In KEGG enrichment of specific classification, the analysis of SZQ, AJH, XQC and SZQ showed the pathways of carbohydrates digestion and absorption with two unique compounds found in both pathways. In comparison, the most enriched pathway in XQC and SZQ was flavonoid biosynthesis, which contributed seven unique compounds. So, the number and type of metabolites vary from cultivar to cultivar based on genetic involvement and their metabolic comparison with another cultivar. Our findings coincide with the aforementioned outcome of Durian [37] and wheat [38].

The study suggests that the quality and quantity of metabolites depend on the physiology and phenology of cultivars. Our results coincide with previous studies that show that the color of Pak choi is influenced by the presence or type of metabolites [39]. In future research, we will investigate the reasons for color variations in the same vegetable under the matching growing conditions concerning physiological, morphological, and metabolic analysis. This will contribute to understanding the phenomenon and behavioral effects in detail and broaden the research exposure.

## 5. Conclusions

The study analyzed the metabolites of seven non-heading Chinese cabbage ‘Pak choi’ cultivars and found significant variations in the metabolites. The primary and secondary metabolites of Pak choi include amino acids, carbohydrates, flavonoids, organic acids, and anthocyanins. The analysis revealed 16 carbohydrates with four classes out of 513 metabolites in 24 groups, with monosaccharides being the most abundant. SZQ had the highest amounts of carbohydrates and compounds, while XQC had the lowest. The metabolites of the violet stem cultivar were Glucono-1, 5-lactone, and glucose, which have health benefits in preventing cardiovascular disease, diabetes, and obesity. The study suggests that the quality and quantity of metabolites depend on the physiology and phenology of cultivars. 

## Figures and Tables

**Table 1:** Phenotypic characters of Pak choi cultivars.

Phenotypic Characters	Pak Choi Cultivars						
	Suzhouqing	Yellowrose	Wutacai	Aijiaohuang	Xiangqingcai	Zicaitai	Ziluolan
**Leaf**							
color	Yellow	Yellow	Dark green	Yellow	Dark green	Yellow	purple
size (cm)	21 × 18	16 × 19	4 × 10	23 × 19	14 × 18	21 × 15	6 × 8
shape	oval	oval	obovate	oval	oval	oval	oval
apex	obtuse	obtuse	obtuse	obtuse	obtuse	obtuse	obtuse
margins	entire	acute	entire	acute	entire	undulate	entire
surface	smooth	entire	smooth	entire	smooth	smooth	smooth
arrangement	alternate	smooth	alternate	smooth	alternate	alternate	alternate
**stem**							
color	light green	light green	light green	light green	light green	pink	light green
**surface**	smooth	smooth	smooth	smooth	smooth	smooth	smooth
**plant height (cm)**	29	32–39	27–38	31	23–37	34–70	20

**Table 2:** The division of carbohydrates is based on leaf colors such as dark green—WTC, purple—ZLL, and yellowish—AJH, SZQ, ZTC, XQC, and YLR.

Category	Monosaccharide	Disaccharide	Oligosaccharide	Polysaccharide
Dark Green	5.91 × 10^5^	1.40 × 10^6^	2.68 × 10^4^	1.05 × 10^6^
Purple	5.10 × 10^5^	6.17 × 10^5^	1.97 × 10^4^	8.59 × 10^5^
Yellowish	5.51 × 10^5^	1.01 × 10^6^	2.33 × 10^4^	9.53 × 10^5^

## Data Availability

The original contributions presented in the study are included in the article/Supplementary Material, further inquiries can be directed to the corresponding author/s.

## Supplementary Materials

The following supporting information can be downloaded at: https://www.mdpi.com/article/10.3390/biology13080568/s1, Supplementary Figure S1. The representation of detected metabolites in a multi-peak plot showed the highly overlapped curves of detected metabolites, indicating the signal stability of mass spectrometry; Supplementary Figure S2. Schematic diagram of the mass spectrometry multi-reaction monitoring mode where the emitting ions correspond to their molecular weight and are selected based on characteristic fragments; Supplementary Figure S3. The most enriched pathway of carbohydrate digestion and absorption in SZQ vs AJH and XQC and SZQ shows the two unique compounds that are D (+)-Glucose and D-Glucose 6-phosphate in these analyses; Supplementary Figure S4. The most enriched KEGG in the analysis of XQC and SZQ was flavonoid biosynthesis in which seven unique compounds such as two Delphinidin, 7,4′-Dihydroxyflavone, Quercetin, Naringenin chalcone, Naringenin 7-O-beta-D-glucoside, and Cyanidi; Table S1. List of known metabolites; Table S2. Up and Down regulation of metabolites; Table S3. List of individual carbohydrates identified in leaves of pak choi cultivars.
