# Supplementary material for: Metabolite Diversity and Carbohydrate Distribution in Brassica campestris ssp. chinensis L. Cultivars: A UPLC-MS/MS Approach
Source: Biology (Basel). 2024 Jul 27;13(8):568. doi: 10.3390/biology13080568 (PMC11351843; doi:10.3390/biology13080568)
Supplement: Supplementary file 1 [file biology-13-00568-s001.zip › Supplementary figures.pdf]

Supplementary Figures

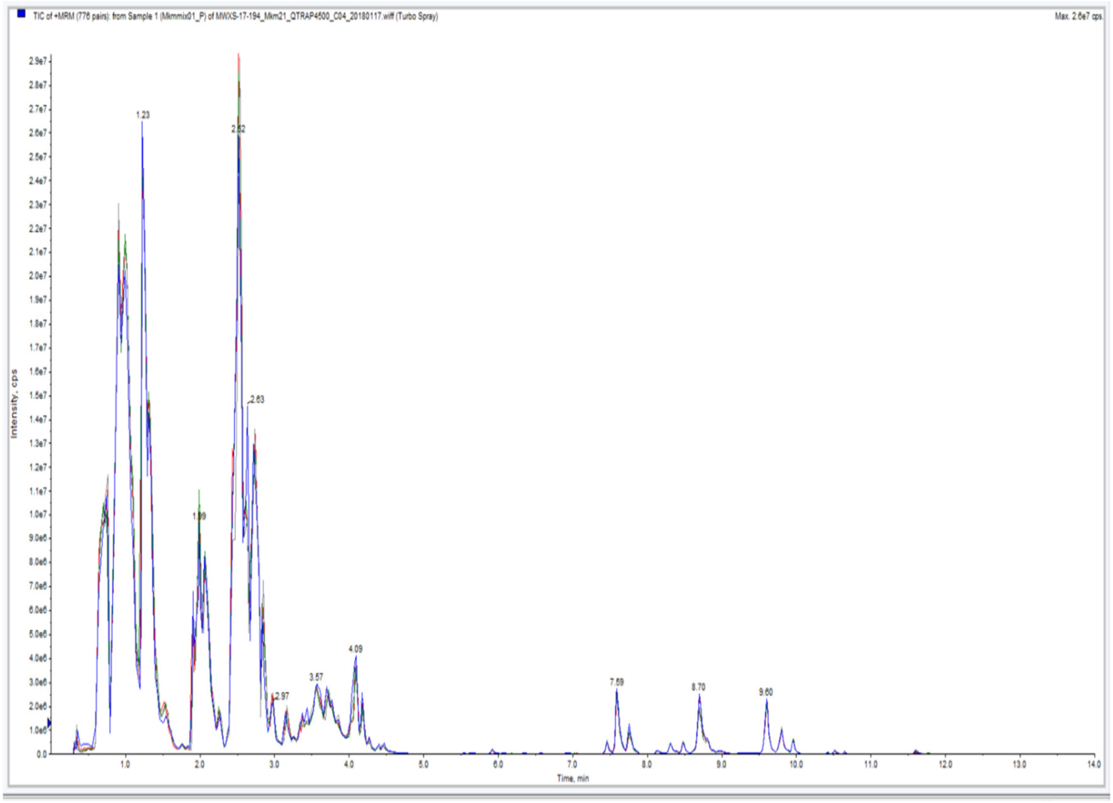

Supplementary Figure S1. The representation of detected metabolites in a multi-peak plot showed the highly overlapped curves of detected metabolites, indicating the signal stability of mass spectrometry.

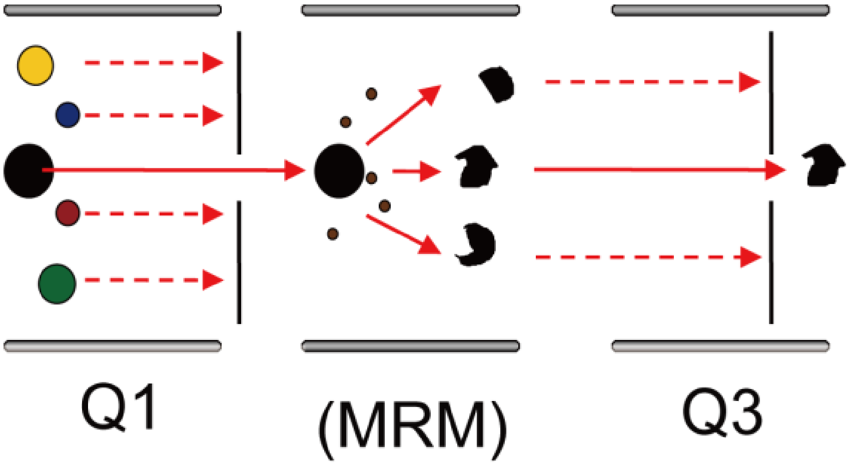

Supplementary Figure S2. Schematic diagram of the mass spectrometry multi-reaction monitoring mode where the emitting ions correspond to their molecular weight and are selected based on characteristic fragments.

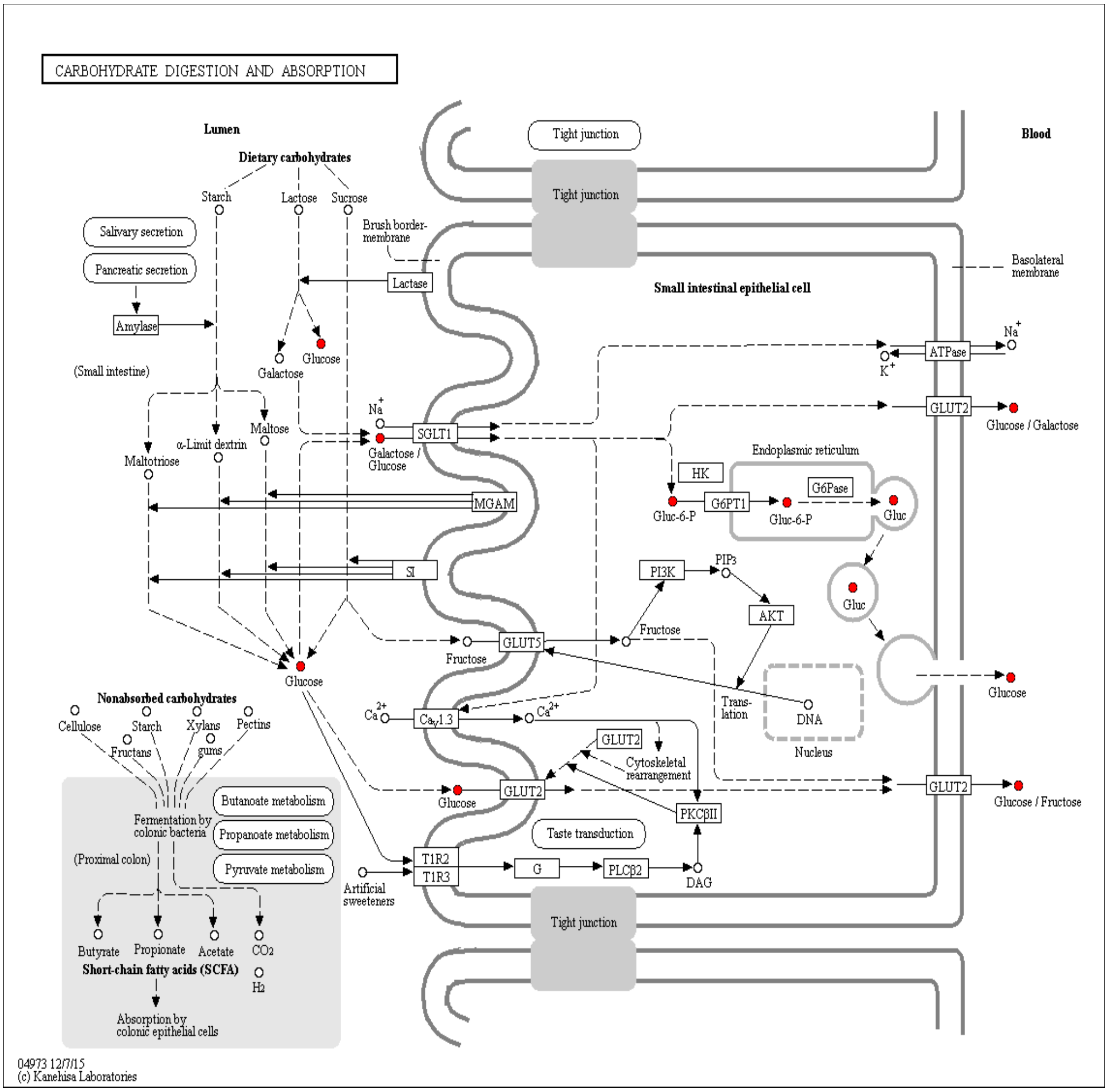

Supplementary Figure S3. The most enriched pathway of carbohydrate digestion and absorption in SZQ vs AJH and XQC and SZQ shows the two unique compounds that are D (+)-Glucose and D-Glucose 6-phosphate in these analyses.

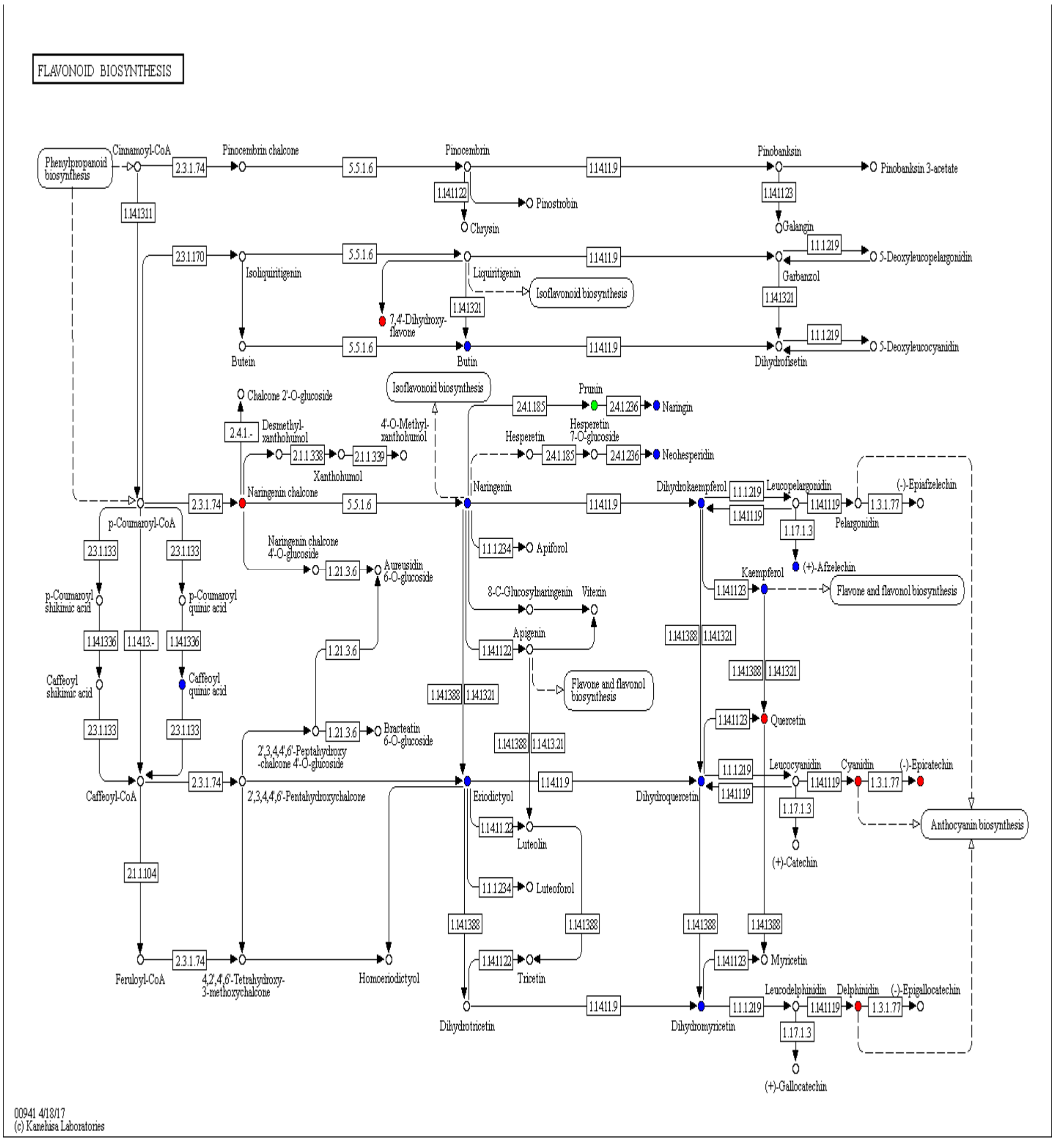

Supplementary Figure S4. The most enriched KEGG in the analysis of XQC and SZQ was flavonoid biosynthesis in which seven unique compounds such as two Delphinidin, 7,4'-Dihydroxyflavone, Quercetin, Naringenin chalcone, Naringenin 7-O-beta-D-glucoside, and Cyanidi.
